# Perilesional Perfusion in Chronic Stroke-Induced Aphasia and Its Response to Behavioral Treatment Interventions

**DOI:** 10.1162/nol_a_00068

**Published:** 2022-05-11

**Authors:** Matthew Walenski, Yufen Chen, Kaitlyn A. Litcofsky, David Caplan, Swathi Kiran, Brenda Rapp, Todd B. Parrish, Cynthia K. Thompson

**Affiliations:** Department of Communication Sciences and Disorders, East Carolina University, Greenville, NC; Center for the Neurobiology of Language Recovery, Northwestern University, Evanston, IL; Department of Radiology, Feinberg School of Medicine, Northwestern University, Evanston, IL; Department of Communication Sciences and Disorders, School of Communication, Northwestern University, Evanston, IL; Massachusetts General Hospital, Department of Neurology, Harvard Medical School, Boston, MA; Department of Speech, Language, and Hearing, College of Health & Rehabilitation, Boston University, Boston, MA; Department of Cognitive Science, Krieger School of Arts & Sciences, Johns Hopkins University, Baltimore, MD; Department of Neurology, Feinberg School of Medicine, Northwestern University, Evanston, IL

**Keywords:** aphasia, stroke, perfusion, MRI, treatment

## Abstract

Stroke-induced alterations in cerebral blood flow (perfusion) may contribute to functional language impairments in chronic aphasia, particularly in perilesional tissue. Abnormal perfusion in this region may also serve as a biomarker for predicting functional improvements with behavioral treatment interventions. Using pseudo-continuous arterial spin labeling in magnetic resonance imaging (MRI), we examined perfusion in chronic aphasia, in perilesional rings in the left hemisphere and their right hemisphere homologues. In the left hemisphere we found a gradient pattern of decreasing perfusion closer to the lesion. The opposite pattern was found in the right hemisphere, with significantly increased perfusion close to the lesion homologue. Perfusion was also increased in the right hemisphere lesion homologue region relative to the surrounding tissue. We next examined changes in perfusion in two groups: one group who underwent MRI scanning before and after three months of a behavioral treatment intervention that led to significant language gains, and a second group who was scanned twice at a three-month interval without a treatment intervention. For both groups, there was no difference in perfusion over time in either the left or the right hemisphere. Moreover, within the treatment group pre-treatment perfusion scores did not predict treatment response; neither did pre-treatment perfusion predict post-treatment language performance. These results indicate that perfusion is chronically abnormal in both hemispheres, but chronically abnormal perfusion did not change in response to our behavioral treatment interventions, and did not predict responsiveness to language treatment.

## INTRODUCTION

Alterations in vascular physiology are common sequela of acute stroke and persist into chronic stages (Fridriksson et al., 2006; Hillis, 2007; Richardson et al., 2011). Evidence from multiple sources suggests that restoration of cerebral blood flow (CBF; the rate at which blood perfuses a neural region) is critically associated with functional recovery due to natural recovery and reperfusion of damaged regions, especially in the hyperacute stage (Hamilton et al., 2011; Saur et al., 2006). However, chronic reductions in CBF (hypoperfusion) in specific regions may contribute to long-term language impairments in individuals with aphasia (Brumm et al., 2010; Love et al., 2002; Richardson et al., 2011; Thompson et al., 2010, 2017).

One region that may be important for understanding chronic functional impairments, and which may therefore also provide a fruitful avenue for recovery, is perilesional tissue. However, what constitutes a “perilesional” region has never been precisely defined (Thompson et al., 2017), and some authors refer to perilesional regions strictly in terms of rings of tissue surrounding the lesion. Here we focus on studies examining such perilesional rings in chronic aphasia, where the extent of the rings identified as perilesional have varied across reports, from 6.88 (left/right, anterior/posterior) or 13.88 mm (inferior/superior) depending on direction (Brumm et al., 2010), to 3–8 mm (Richardson et al., 2011), 3–15 mm (Fridriksson et al., 2012), 0–6 mm (Thompson et al., 2017), 0–5 mm (Boukrina et al., 2019), and 0–3 mm (Abbott et al., 2021).

Despite variable definitions of how far perilesional tissue extends, multiple results indicate that perfusion in perilesional rings is abnormally low relative to regions of the ipsilesional hemisphere that are further from the lesion (Boukrina et al., 2019; Fridriksson et al., 2012; Thompson et al., 2017). Fridriksson and colleagues (2012) report a gradient pattern, with significant reductions in perfusion across multiple successive 3 mm rings surrounding the lesion—perfusion in a 3–6 mm ring was lower than in a 6–9 mm ring, which was in turn lower than in a 9–12 mm ring, again in turn lower than in a 12–15 mm ring. Perfusion in perilesional rings is also consistently found to be abnormally low relative to undamaged homologue regions in the contralesional hemisphere (Brumm et al., 2010; Richardson et al., 2011; Thompson et al., 2017), though only marginally so in one study (Boukrina et al., 2019).

Importantly, reperfusion of hypoperfused tissue has been shown to provide a notable avenue for recovery of function, in animals and humans in the acute stage of language impairments (Chollet et al., 1991; Hillis, 2005; Lee & van Donkelaar, 1995), and treatment interventions for motor impairments in chronic stroke have led to improved perfusion in motor areas (Hara et al., 2013; Könönen et al., 2005). In chronic aphasia as well, treatment with transcranial magnetic stimulation has led to improved language function and a leftward (i.e., ipsilesional) shift in perfusion in specific language areas of interest (notably BA44, with right hemisphere stimulation), measured with single photon-emission computed tomography (SPECT; Hara et al., 2015). However, we are aware of only one prior study in chronic aphasia that measured changes in perfusion in perilesional rings over time. In this natural recovery study, no changes in perilesional perfusion between subacute (1–5 weeks) and chronic periods were found (Boukrina et al., 2019). We are not aware of any prior studies of chronic aphasia that have found changes in perfusion in perilesional rings in response to behavioral treatment interventions.

Prior studies that examined the relationship between pre-treatment perfusion in perilesional rings and recovery of function in chronic aphasia produced mixed results. Boukrina et al. (2019) examined longitudinal recovery (without treatment) between subacute (at 5 weeks post-stroke) and chronic (greater than 3 months post) periods in 15 participants with single-word reading deficits, and found that subacute perilesional perfusion levels in the left hemisphere were not predictive of subsequent (chronic) word reading accuracy. Similarly, a study of 30 chronic-state patients with anomia (minimum 6 months post-stroke) reported that pre-treatment cortical perfusion in perilesional cortex (3–15 mm from the lesion) was not associated with changes in correct naming in response to treatment (Fridriksson et al., 2012). However, Boukrina et al. (2019) reported a negative correlation: Increased subacute perfusion in right hemisphere (RH) homologous perilesional rings was associated with worse chronic word reading accuracy.

Note that while perfusion in the unlesioned right hemisphere has been used as a “normal” standard of comparison for left hemisphere (LH) perilesional rings, we are aware of only two studies that report comparisons across RH homologue regions. In one study, no difference in perfusion levels was seen between an RH homologue ring (3–8 mm) and the distal remainder of the right hemisphere (Richardson et al., 2011). In another, no difference in RH perfusion levels was seen between 0–6 mm, 6–12 mm, and 12–18 mm homologue rings (Thompson et al., 2017).

Here we focus on three main questions. In experiment 1, we examine the status of perfusion in chronic aphasia in LH perilesional rings and their RH counterparts. In experiment 2, we examine the extent to which perilesional perfusion levels in these rings change in response to behavioral language interventions. In experiment 2 we also examine whether the pre-treatment perilesional perfusion levels in the left hemisphere, or their RH homologues, predict a treatment-modulated improvement in language. Based on prior results, we expect LH perilesional perfusion to be abnormally low, relative both to RH homologue regions and to more distal LH tissue. In addition, perfusion levels in these rings may not change in response to our behavioral treatment interventions. Finally, previous results suggest that perilesional hypoperfusion should not predict treatment efficacy, whereas there may be a maladaptive relationship for RH homologue rings, such that increased perfusion predicts a worse treatment outcome.

## EXPERIMENT 1

### Method

#### Participants

We tested 71 participants with aphasia subsequent to a single LH ischemic stroke (Table 1A). Participants with different aphasias were recruited from three research sites, Northwestern University (agrammatism; *n* = 17), Boston University (anomia; *n* = 30), and Johns Hopkins University (dysgraphia; *n* = 24), as part of a large-scale Clinical Research Center funded by the NIDCD (National Institute on Deafness and Other Communication Disorders). The study was approved by the Institutional Review Boards of all three universities, and all participants provided informed consent. Note that pre-treatment perfusion data from 35 of these participants was previously reported (Thompson et al., 2017). Note that an additional 10 participants were tested, but their data were excluded due to artifacts (see Data Processing, below). The demographic details here include only the 71 participants in the final data set.

**Table 1.** Participant information (mean and standard deviation)

	Age (years)	Sex	Education (years)	Months post-stroke	WAB-AQ
(A)					
Aphasia (*n* = 71)	58.3 (10.3)	48 M / 23 F	15.8 (2.1)	62.1 (50.4)	68.1 (22.2)

(B)					
Treatment (*n* = 45)	59.3 (10.0)	30 M / 15 F	15.8 (2.2)	58.5 (43.8)	65.0 (24.1)
Control (*n* = 16)	56.8 (12.3)	12 M / 4 F	15.8 (2.0)	58.4 (50.9)	74.1 (18.0)

*Note*. WAB-AQ = Western Aphasia Battery—Aphasia Quotient (Kertesz, 2006).

Participants were all in the chronic stage of aphasia, and were at least eight months post-stroke-onset (*M* = 62.1 months, *SD* = 50.4, range: 8–209 months). All participants were (premorbid) right-handed native English speakers (48 M, 23 F; age: range = 39–79 yr, *M* = 58.8, *SD* = 10.3; education: *M* = 15.8 yr, *SD* = 2.1); 6 of the participants with anomia were bilingual, though English was their dominant language. Participants passed vision and hearing screenings (pure-tone audiometric screening at 40 dB, 1000 Hz), and had no other diagnosed brain disorders and no history of drug or alcohol abuse.

The characterization, diagnosis, and overall severity of aphasia was based on administration of the Western Aphasia Battery—Revised (WAB-R; Kertesz, 2006); WAB-AQ (Aphasia Quotient) scores ranged from 11.7 to 97.5 (*M* = 68.1, *SD* = 22.2); and a battery of standard language tests, which included measures of spoken and written comprehension and production of words and sentences. Note that while the WAB-AQ scores for some participants (*n* = 5, 1 with anomia and 4 with dysgraphia) were above the standard cutoff for a diagnosis of aphasia (93.8), our participants with high WAB-AQ scores were nevertheless characterized as aphasic based on their scores on our standard language battery.

Our standard language battery consisted of language tasks chosen to identify individuals with agrammatism, anomia, or dysgraphia. Single word production and comprehension were tested using 26 items from the Confrontation Naming and Auditory Comprehension subtests of the Northwestern Naming Battery (NNB; Thompson & Weintraub, 2014) (10 low frequency nouns from the “Other” category on the NNB and 16 verbs). Word production performance ranged from 0% to 100% (*M* = 58%, *SD* = 31%); word comprehension performance ranged from 24% to 97% (*M* = 75%, *SD* = 17%). We used the Psycholinguistic Assessments of Language Processing in Aphasia (PALPA; Kay et al., 1996) to evaluate spelling-to-dictation of words with high and low frequency (subtest 40) (*M* = 36%, *SD* = 31%, range: 0% to 98%). Finally, we used the Sentence Production Priming Test (SPPT) and the Sentence Comprehension Test (SCT) from the Northwestern Assessment of Verbs and Sentences (NAVS; Thompson, 2011) to evaluate production and comprehension of sentences of different syntactic complexity. Each test includes 30 items to test canonical and noncanonical structures. Performance on sentence comprehension for noncanonical sentences ranged from 27% to 100% (*M* = 64%, *SD* = 20%), and for canonical sentences performance ranged from 33% to 100% (*M* = 79%, *SD* = 20%). For sentence production, performance for noncanonical sentences ranged from 0% to 100% (*M* = 33%, *SD* = 34%), and for canonical sentences performance ranged from 0% to 100% (*M* = 49%, *SD* = 37%).

#### Data Acquisition

Images were collected on four different 3.0T systems: a Siemens TIM Trio with a 32-channel head coil (Northwestern University), a Siemens Prisma with a 64-channel head/neck coil (Northwestern University), a Skyra with 20-channel head/neck coil (Boston University), and a Philips Intera with a 32-channel head coil (Johns Hopkins University). Prior to the study, a study team member acted as a travel phantom to all sites, where trial data were collected and inspected to ensure identical imaging parameters.

Resting CBF maps were collected using a pseudo-continuous arterial spin labeling (pCASL) sequence (Dai et al., 2008) with two-dimensional gradient echo-planar imaging (EPI) readout: field of view (FOV) = 220 mm, in-plane resolution = 3.4 × 3.4 mm^2^, 25 slices, thickness = 4 mm with 1 mm gap, TE/TR = 11 ms / 4,500 ms. The labeling plane was situated 90 mm below the center of the imaging volume, and labeling pulses were applied for 1.5 s. The post labeling delay was set to 1,900 ms to balance between potential slow flow and adequate signal-to-noise ratio (Alsop et al., 2015). Sixty pairs of interleaved control and tag images were acquired for signal averaging. In addition to the ASL scan, high resolution T1-weighted anatomical images were acquired using an MPRAGE (magnetization-prepared rapid acquisition with gradient echo) sequence (Mugler & Brookeman, 1990): FOV = 256 mm, TE/TR/TI = 2.91 ms / 2300 ms / 900 ms, 176 sagittal slices, resolution 1 mm^3^.

#### Data Processing

Perfusion-weighted images from the pCASL scan were processed at a single site by an expert in perfusion imaging, using a pipeline incorporating commands from Statistical Parametric Mapping (SPM8; Wellcome Trust Centre for Neuroimaging, London, UK), and code developed in-house with Matlab R2013a (Mathworks, Natwick, MA), and implemented on the Northwestern University Neuroimaging Data Archive (NUNDA; Alpert et al., 2016). Briefly, the raw EPI images were aligned to the first image of the time series to extract six motion-related measures for the time series. The motion parameters and signal from voxels containing 99% cerebrospinal fluid were regressed out of the time series to remove motion-related and physiological fluctuations in the signal (Wang, 2012). Perfusion weighted time series were generated using pairwise subtraction, and outliers were removed based on the following criteria (Wang et al., 2008): (a) translation greater than 0.8 mm; (b) rotation greater than 0.8°; and (c) global signal or noise greater than 2 times the standard deviation. Based on these criteria, an average of 7 pairs of images was discarded from each ASL scan for participants with agrammatism or anomia, and 8 pairs of images for participants with dysgraphia.

After processing, participant data were visually inspected and were assessed based on summary statistics including the number of images discarded due to motion, raw signal-to-noise ratio of mean perfusion weighted images, and global CBF values. Data sets with global CBF values less than 20 ml / 100 g / min or discarded images more than 2 times the standard deviation + mean number of discard images were labeled as unusable and excluded from final analysis. This resulted in the exclusion of data from 10 participants (2 with agrammatism, 5 with anomia, and 3 with dysgraphia).

The final perfusion weighted time series were then converted into quantitative flow (*f*) maps in units of ml / 100 g / min using the following equation:

$$f=\frac{\lambda \cdot \Delta M}{2\alpha M_{0}\cdot T_{1b}\cdot \left(e^{-PLD/T_{1b}}-e^{-\left(\tau +PLD\right)/T_{1b}}\right)},$$

where *λ* is the blood/tissue partition coefficient = 0.9 ml / g (Herscovitch & Raichle, 1985), Δ*M* is the perfusion-weighted signal, *α* is the inversion efficiency = 0.85 (Dai et al., 2008), *M*_0_ is the equilibrium signal of tissue, PLD is the post-labeling delay, *τ* the labeling duration, and *T*_1*b*_ is the *T*_1_ of blood = 1,664 ms (Lu et al., 2004).

Due to the low resolution of CBF maps, partial volume effects are prominent and need to be corrected before any further analysis. This was implemented as another NUNDA pipeline based on the following equation derived from positron emission tomography CBF studies (Du et al., 2006):

$$f_{GM}=\frac{f_{\text{uncorr}}-P_{WM}\cdot f_{WM}}{P_{GM}},$$

where *f*_*uncorr*_ is the uncorrected flow value, *P*_*GM*_ and *P*_*WM*_ denote gray and white matter probability in the voxel, extracted from tissue segmentation of the high-resolution anatomical image, and *f*_*GM*_ and *f*_*WM*_ are the corresponding tissue-specific flow values. *f*_*WM*_ was extracted from voxels containing 99% white matter. To minimize artifactually high CBF due to division by small numbers, the above calculation was limited to voxels containing at least 30% gray matter (Chen et al., 2011; Hu et al., 2010). The partial volume corrected CBF maps were then spatially normalized to MNI (Montreal Neurological Institute) space using the transformation matrix calculated from the high-resolution anatomical image after co-registering the ASL data to the native T1 scan. We removed any voxels that were left with negative values after these processing steps, as these could only reflect noise in the data.

Finally, as CBF is a physiological parameter that fluctuates with many factors such as vasoactive agents in food, beverages, and drugs, and varies widely between subjects (Clement et al., 2018), we normalized all CBF values to the mean CBF of each individual’s right occipital lobe region, in order to reduce between-subject variation. We based the definition of the right occipital lobe cortical region on the Harvard Oxford atlas (https://neurovault.org/collections/262/), processed as above for the perilesional regions of interest (ROIs). Note that this choice assumes that CBF in this region is not compromised by an LH stroke resulting in aphasia.

#### Lesion Volume

Lesion volume was derived from lesion maps, developed by manual drawings measured using MRIcron software (https://www.nitrc.org/projects/mricron; Rorden & Brett, 2000), traced (and confirmed) by lab members with extensive experience with MRI and lesion tracing. To delineate the borders of necrotic tissue in each patient, we first determined intensity measures for white and grey matter (WM and GM, respectively) in the contralateral (right) hemisphere for each axial slice. The minimum right hemisphere WM intensity was determined. Left hemisphere lesioned tissue, on each slice, was drawn using the pen tool of MRIcron. Then the minimum WM intensity from the right hemisphere was applied to the outlined area in the left hemisphere using the intensity filter function, to avoid including voxels in the lesion mask that were potentially white matter. Additional manual correction was applied using lesion outlines in multiple corresponding coronal and sagittal views. Total lesion volume was calculated by summing the number of lesioned voxels in the left hemisphere for each participant. In our analyses the size of each voxel was 1 mm^3^ and therefore lesion volume is reported in mm^3^.

#### Regions of Interest

**Figure 1.**
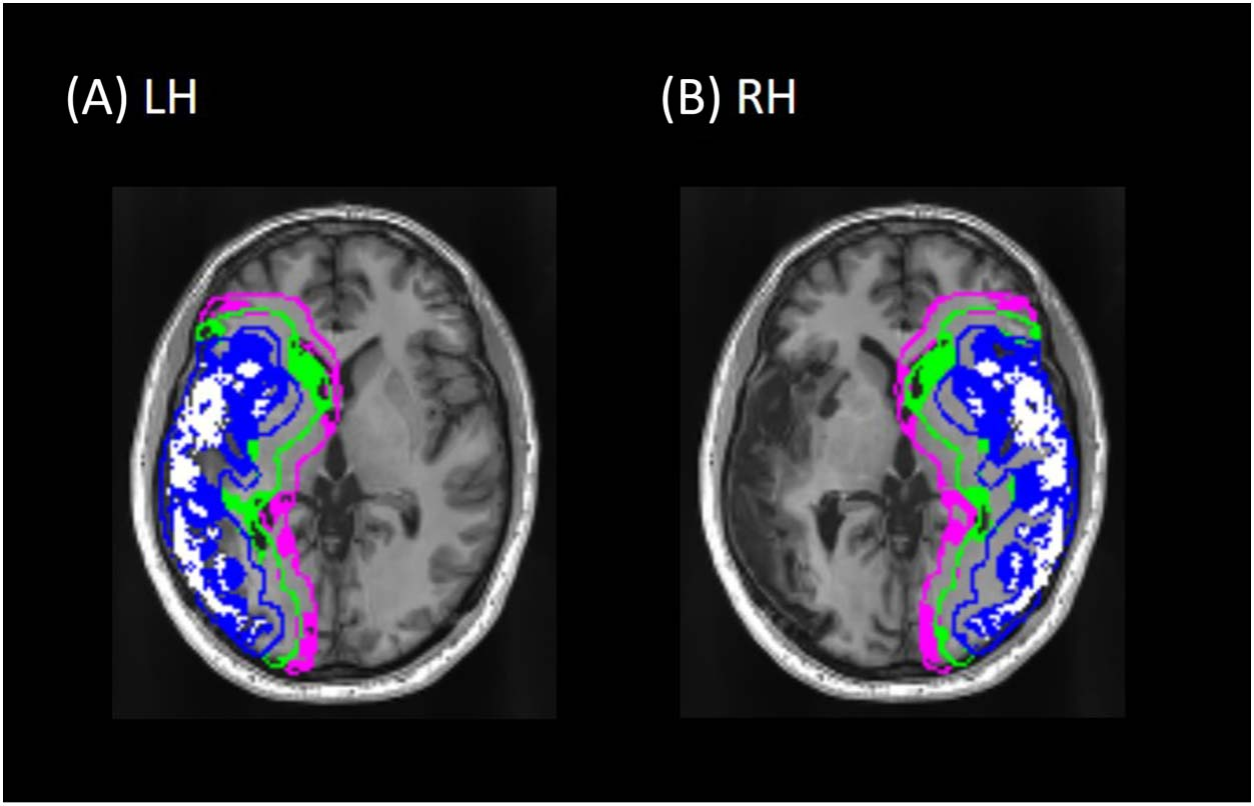
An axial slice from a representative participant showing the lesion and perilesional ROI masks for (A) the left hemisphere (LH) and (B) the right hemisphere (RH) homologue regions. The lesion and its homologue are in white; 0–6 mm ring in blue, 6–12 mm in green, and 12–18 mm in pink. The rings are outlined in their respective colors to show the boundary of each ring; the filled-in portions of each ring reflect gray matter within each ring (only gray matter voxels within each ring were used to calculate perfusion). Note that the rings reflect distance from the lesion in three dimensions—that is, portions of a ring that are visible in this slice may reflect lesioned tissue that is above or below the plane of this image slice, making the rings appear larger than expected for the lesion visible in the image.

Regions of interest and their RH homologues were created by first identifying the ROI of the lesion in the left hemisphere (taking the homologous RH region), and then dilating the lesion to 6 mm, to 12 mm, and to 18 mm beyond its boundaries and subtracting the original lesion and smaller perilesional volumes for each ROI (Figure 1). Thus we created three perilesional rings that were 0–6 mm, 6–12 mm, and 12–18 mm from the lesion, which were confined to the LH grey matter. The size of the rings was chosen to be consistent with our previous work (Thompson et al., 2017). Mean CBF within each ROI was only computed from voxels with 30% or more gray matter, as division by a small number (*P*_*GM*_, see partial volume correction equation above) yields unrealistically high CBF values. In addition to correcting for partial volume, the ROIs also accounted for the lesion mask (voxels where the lesion value is set to 1 were excluded from the analysis) and the FOV of the perfusion scan (an FOV mask was created to exclude all voxels not covered by the perfusion scan).

#### Data Analysis

Data from the full pre-treatment cohort (*n* = 71) were analyzed with mixed effects linear regression models (proc glimmix; SAS 9.4 for Windows; SAS Institute, 2012) with a random effect of participant on the intercept and fixed effects of Hemisphere (LH, RH), Ring (0–6 mm, 6–12 mm, 12–18 mm), and their interaction. We included additional random effects by participant if their inclusion improved model fit, based on the Bayesian information criteria (BIC). Reductions to BIC greater than 10 (as a chi-square with 1 degree of freedom) were considered an improved fit. Based on these criteria, Hemisphere was included as a random effect (BIC changed from −597.17 in the intercept only model to −642.58 when Hemisphere was added); but Ring (BIC = −636.05) and the Hemisphere by Ring interaction (BIC = −609.82) did not improve model fit, and so were included as fixed effects only.

We also included several additional predictor variables as fixed effects, to account for potential sources of variability in the data. We included participant age and sex, and given that our participants were tested on 4 different scanners, we included scanner as well. We also included (square root transformed) lesion volume. The transformation was applied to remove the positive skew in the distribution of lesion volumes.

Follow-up contrasts were assessed for ring differences within each hemisphere and for the same rings across hemispheres. For these contrasts we report the regression coefficient (*B*) with its standard error, the *t* test of the difference, and the 95% confidence interval. We applied the Benjamini-Hochberg false discovery rate (FDR) correction (Benjamini & Hochberg, 1995) for multiple comparisons (with an FDR of 0.05) to these contrasts, and report *q* values (two-tailed) instead of *p* values.

We assessed the comparison of perfusion in the RH lesion homologue against the 0–6 mm RH ring with an additional regression model, with random effects of participant on the intercept, and a fixed effect of Region (lesion homologue vs. 0–6 mm ring). Adding Region as a random effect did not improve model fit (BIC for intercept only model = −132.64; BIC with region added as random effect = −126.25), so it was included in the model as a fixed effect only. Square root transformed lesion volume, scanner, age, and sex were included as additional independent predictor variables.

All models were fit with an unstructured covariance matrix. We report *F* and *p* values from Type III tests of the main effects and interactions of these factors. Degrees of freedom were computed using the Satterthwaite approximation (note that these degrees of freedom are calculated based on the variance, and are not the same as in ANOVA). Significance of all comparisons was assessed with α = 0.05. All of the reported *p* values are two-tailed.

### Results

**Figure 2.**
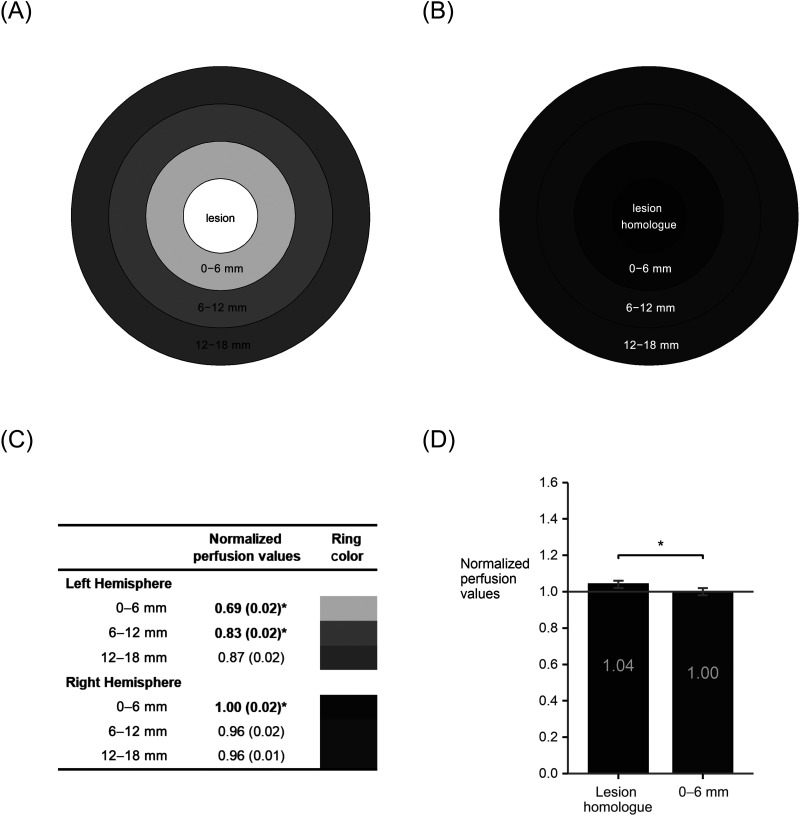
Pre-treatment perilesional perfusion in left hemisphere (LH) perilesional rings (A) and their right hemisphere (RH) homologues (B). Ring color is scaled to perfusion values, with lighter grays for lower perfusion values and darker grays for higher perfusion values. A table (C) shows normalized perfusion values and standard error for each perilesional ring and its RH homologue, with scaled colors corresponding to the rings in (A) and (B). Values with * and in bold indicate that the smaller ring is significantly different from the next larger ring within the same hemisphere (*q* < 0.05). For all three rings, perfusion was significantly lower in the LH than the RH (*q* < 0.05; see text). An additional analysis (D) revealed significantly elevated perfusion for lesion-homologous tissue in the right hemisphere relative to the RH 0–6 mm ring (*p* < 0.0001). All perfusion values are normalized to a right occipital cortical ROI.

We examined CBF in perilesional tissue in all participants (*n* = 71) prior to treatment (Figure 2). There were significant main effects of Hemisphere (lower perfusion in LH than RH; *F*(1, 70) = 177.16, *p* < 0.0001), and Ring (*F*(2, 70) = 18.5, *p* < 0.0001), but also a significant Hemisphere × Ring interaction (*F*(2, 140) = 148.55, *p* < 0.0001). The effects of lesion volume (*F*(1, 64) = 0.04, *p* = 0.83), scanner (*F*(3, 64) = 1.0, *p* = 0.40), age (*F*(1, 64) = 0.2, *p* = 0.66), and sex (*F*(1, 64) = 0.00, *p* = 0.95) were not significant.

Follow-up contrasts confirmed that within the left hemisphere, perfusion was significantly lower in the 0–6 mm perilesional ring relative to the 6–12 mm (*B* = −0.14, *SE* = 0.01, *t*(170) = 13.1, 95% CI [−0.16, −0.12], *q* = 0.0001); perfusion in the 6–12 mm ring was also significantly lower than in the 12–18 mm ring (*B* = −0.04, *SE* = 0.01, *t*(176) = 4.28, 95% CI [−0.06, −0.02], *q* = 0.0001). However, the opposite pattern was found within the right hemisphere, with significantly greater perfusion in the 0–6 mm ring relative to the 6–12 mm ring (*B* = 0.05, *SE* = 0.01, *t*(170) = 4.51, 95% CI [0.03, 0.07], *q* = 0.0001), but the 6–12 mm ring did not differ from the 12–18 mm ring (*B* = 0.005, *SE* = 0.01, *t*(176) = 0.53, 95% CI [−0.01, 0.03], *q* = 0.6).

Across hemispheres, contrasts revealed that perfusion in the left hemisphere was significantly lower than perfusion in the right hemisphere, in the 0–6 mm ring (*B* = −0.31, *SE* = 0.016, *t*(121) = 20.15, 95% CI [−0.34, −0.28], *q* = 0.0001), as well as the 6–12 mm ring (*B* = −0.13, *SE* = 0.015, *t*(121) = 8.29, 95% CI [−0.16, −0.10], *q* = 0.0001), and the 12–18 mm ring (*B* = −0.09, *SE* = 0.015, *t*(121) = 5.83, 95% CI [−0.12, −0.06], *q* = 0.0001).

Perfusion in the RH lesion homologue region was significantly elevated beyond the elevated perfusion found in the 0–6 mm RH perilesional ring (homologue *M* = 1.04, *SE* = 0.02; 0–6 mm ring *M* = 1.00, *SE* = 0.02; *F*(1, 70) = 17.25, *p* = < 0.0001; Figure 2D). Effects of lesion volume, scanner, age, and sex were all nonsignificant (all *p*s > 0.39).

### Discussion

We examined blood flow in a cohort of individuals with chronic aphasia prior to treatment, in three LH perilesional rings and their RH homologues, as well as in the RH lesion homologue region.

In the absence of treatment, the results for all three perilesional rings revealed that perfusion in the damaged left hemisphere was lower than in the undamaged right hemisphere, consistent with previous findings (Boukrina et al., 2019; Brumm et al., 2010; Richardson et al., 2011; Thompson et al., 2017). Within the left hemisphere as well, tissue closer to the lesion was reduced in perfusion relative to more distant areas—for the 0–6 mm ring compared to the 6–12 mm ring, and for 6–12 mm vs. 12–18 mm. This is again consistent with prior findings (Abbott et al., 2021; Boukrina et al., 2019; Fridriksson et al., 2012; Thompson et al., 2017), but is most consistent with one prior study that reports a similar gradient across successive 3 mm rings (Fridriksson et al., 2012).

The finding that there is a similar gradient pattern across different size rings suggests that the gradient may be continuous—that is, perfusion may gradually increase the further one gets from the lesion. Dividing such a smoothly changing variable into discrete chunks would yield effects consistent with those we and others have observed. It is not clear how steep such a gradient may be (i.e., how quickly it changes with distance from the lesion, if indeed it changes linearly), or how uniform it may be (i.e., the gradient may be steeper or shallower in different directions).

In the right (contralesional) hemisphere we observed that perfusion in the lesion homologue region was elevated relative to the tissue surrounding it (0–6 mm homologue ring). Cerebral blood flow was also elevated in the innermost perilesional homologue ring (0–6 mm) relative to the next more distal ring (6–12 mm). There was no difference beyond that (6–12 mm vs. 12–18 mm). Thus the right hemisphere seems to capture an inverse echo of the lesion, in a region of locally increased perfusion, extending to the immediate perilesional space. This effect was not seen in prior studies, which report no difference between RH perilesional homologue regions (Richardson et al., 2011; Thompson et al., 2017). This may be because the effect was relatively small (0–6 mm vs. 6–12 mm, Hedge’s *g*_av_ = 0.30), and prior studies had fewer participants. These prior studies also did not examine perfusion in the RH lesion homologue region.

These results are unlikely to reflect individual variability due to factors known (or suspected) to modify perfusion measurements. These perfusion modifiers, related to physiology (e.g., age, sex, blood pressure), blood components (e.g., gasses such as O_2_ and CO_2_, cholesterol), mental state (e.g., mood, stress, anxiety), and drugs (e.g., caffeine and other legal or illegal substances) can introduce large sources of inter-individual variability (Clement et al., 2018). We took several steps to mitigate the impact of these factors. First, we asked participants to maintain their normal caffeine levels prior to testing, to avoid issues due to unusual drowsiness, alertness, or other effects on mood or cognition. Most important, however, because it was not possible for us to obtain objective measures for most of these factors, we normalized each participant’s data to a region of their brain that was most likely to have been undisturbed by their lesion. We also included age and sex in the analyses, to further reduce the impact of individual differences due to these factors, though we note that these factors were not significant in our analyses. The findings also do not appear to reflect differences in the scanners that were used to acquire the data, or differences in lesion volume across participants, as neither factor was a significant predictor of perfusion values in any analysis. Therefore, we argue that our results are unlikely to reflect random variation across participants due to these (or other) perfusion modifiers.

It has been previously suggested that chronically abnormal CBF in aphasia may reflect an autoregulatory change to altered LH vasculature, particularly with respect to regions that appear to be hyperperfused (Thompson et al., 2017). That is, blood typically directed automatically to the LH middle cerebral artery could be shifted elsewhere, for example, to the right middle cerebral artery, leading to increased perfusion in regions supplied by that artery. While autoregulatory disturbances in ischemic stroke are typically of short duration, such disturbances have been observed in chronic stroke (Kunz & Iadecola, 2008). Consistent with this argument, contralesional hyperperfusion has been observed in a small number of prior studies in cortical and subcortical regions (Miao et al., 2018; Thompson et al., 2017; Wang et al., 2019). However, the current findings suggest that there may be a more specific pattern that persists in chronic aphasia, with a regional increase in perfusion in the undamaged hemisphere that mirrors the lesion homologue and proximate perilesional tissue.

## EXPERIMENT 2

### Method

#### Participants

The same participants from experiment 1 were randomly assigned either to a treatment group or to a control group that did not receive treatment. Randomization of participants to the treatment or control group occurred independently at each recruitment site, and was carried out by a staff member other than the person who performed the language assessment. The principal investigator(s) at each recruitment site were not blinded to allocation of participants. Individuals in the treatment (*n* = 45: 10 with agrammatism, 19 with anomia, 16 with dysgraphia) and control (*n* = 16: 5 from Northwestern University, 5 from Boston University, 6 from Johns Hopkins University) groups (Table 1B) were scanned at baseline (pre-treatment) and again 12 weeks later (after the end of treatment for the treatment group). Note that 8 participants did not complete their second scan, and so are not included here. Data from an additional 2 participants were excluded after data processing, due to excessive artifacts in the data, based on the same criteria described above. The treatment and control groups did not differ with respect to age (2-sample unequal variance *t* test: *t*(22) = 0.74, *p* = 0.47), proportion of male and female participants (χ^2^ = 0.38, *p* = 0.54), years of education (*t*(29) = 0.07, *p* = 0.94), or months post-stroke onset (*t*(23) = 0.01, *p* = 0.99). The participants in the treatment group had lower WAB-AQ scores than the participants in the control group (treatment Mean: 65.0, control Mean: 74.1), but this difference was not significant (*t*(35) = 1.58, *p* = 0.12).

#### Data Acquisition

Data in each scan was acquired as described for experiment 1. Pre-treatment and post-treatment scans were taken approximately 3 months apart. Participants were asked to maintain their normal caffeine levels for each scan, which were conducted at the same time of day across sessions for each participant.

#### Data Processing, Lesion Volume, ROI Calculation

These were all the same as for experiment 1.

#### Treatment Interventions

Different treatment interventions were given to participants at each of the three sites, corresponding to their aphasia profile. Treatment at each site was administered in 90-minute sessions twice per week for 12 weeks. At all sites, treatment was provided by trained research assistants and monitored for fidelity, with an independent observer scoring half of the treatment sessions for adherence to the treatment protocol. All sites also monitored treatment progress through weekly probe tasks that assessed trained and untrained items. Different tasks were used at each site to assess pre-treatment and post-treatment accuracy on the trained items. These tasks were sentence production and comprehension (agrammatism), spelling-to-dictation (dysgraphia), or picture naming (anomia). Because different treatments and assessment tasks were used at each site, we computed treatment efficacy for each participant in terms of the change in their percentage correct scores from pre-treatment to post-treatment. Details of the specific treatment and treatment schedule for participants at each site follow. Additional details are provided elsewhere for agrammatism (Barbieri et al., 2019), anomia (Gilmore et al., 2020; Johnson et al., 2019), and dysgraphia (Shea et al., 2020; Wiley & Rapp, 2019).

Participants with agrammatism were given Treatment of Underlying Forms (Barbieri et al., 2019; Thompson & Shapiro, 2005), a treatment approach that uses a series of metalinguistic steps focused on verbs, verb argument structure, and thematic-syntactic mapping in active and passive sentences to improve sentence comprehension and production. Participants were trained on 10 different semantically reversible passive sentences (e.g., *The boy was shaved by the man in the barbershop*). Treatment efficacy was assessed with computer-based sentence production priming (production) and sentence-picture matching (comprehension) tasks. The average of the scores from the two tasks was used to quantify treatment efficacy.

Participants with dysgraphia were given treatment for spelling deficits using an individually tailored set of words for each participant (Shea et al., 2020; Wiley & Rapp, 2019). For each individual we selected forty training words such that baseline letter accuracy on each word was between 25% and 85%. The treatment consisted of a spell-study-spell technique. Treatment efficacy was assessed with a spelling-to-dictation task.

Participants with anomia were given a treatment for anomia using typicality-based semantic features analysis, in which either typical (e.g., *pigeon*) or atypical (e.g., *penguin*) exemplars of a certain semantic category (e.g., birds) were trained (Gilmore et al., 2020; Johnson et al., 2019). Treatment efficacy was assessed with a confrontation naming test consisting of 180 items (in random order) from five semantic categories (i.e., birds, vegetables, fruit, clothing, and furniture) including 36 exemplars of each category, and further divided into half-categories by typicality (i.e., 18 typical; 18 atypical).

#### Data Analysis

We assessed changes in perfusion over time in treated and untreated groups with the same approach as in experiment 1, using mixed effects linear regression models (SAS 9.4, proc glimmix) with a random effect of participant on the intercept and fixed effects of Hemisphere (LH, RH), Ring (0–6 mm, 6–12 mm, 12–18 mm), Group (treatment, control), Time (pre-treatment, post-treatment), and their interactions.

We included additional random effects by participant based on whether their inclusion improved model fit, based on the BIC. Reductions to BIC greater than 10 (as a chi-square with 1 degree of freedom) were considered an improved fit. Based on these criteria, Hemisphere was included as a random effect (BIC changed from −842.61 in the intercept only model to −861.57 when Hemisphere was added); but Ring (BIC = −837.81) did not and so was included as a fixed effect only. Time also improved model fit (BIC = −1,161.48) and so was included as a random effect. Interactions were not considered for inclusion as random effects, given that they did not improve model fit in experiment 1, and that there was a much larger set of interactions here. As in experiment 1, (square root transformed) lesion volume, scanner, age, and sex were included as additional independent predictor variables.

Significance of the change in behavioral treatment scores (post- minus pre- difference scores) was assessed with a *t* test of this difference against 0. Finally, we used regression models to examine whether pre-treatment perfusion values in any of our 3 left hemisphere or 3 right hemisphere-homologue perilesional rings predicted either changes in behavioral treatment scores or post-treatment scores (in separate models), adjusting in all cases for the (square root transformed) lesion volume, scanner, age, and sex, and correcting for multiple comparisons using the Benjamini-Hochberg FDR correction (Benjamini & Hochberg, 1995), with an FDR of 0.05.

### Results

We examined changes in perfusion in the participants who were scanned at both time points, comprising a treatment group (*n* = 45) and an untreated control group with aphasia (*n* = 16) (Table 2). Consistent with the results from the full group (above), there were significant main effects of Hemisphere (left lower than right: *F*(1, 59) = 136.14, *p* < 0.0001), Ring (0–6 mm vs. 6–12 mm vs. 12–18 mm: (*F*(2, 531) = 51.29, *p* = < 0.0001), and a significant Hemisphere by Ring interaction (rings closer to the lesion are increasingly hypoperfused in the left hemisphere, 0–6 mm hyperperfused in the right: *F*(2, 531) = 135.42, *p* < 0.0001). The main effect of Time was not significant (*F*(1, 59) = 0.57, *p* = 0.46). The effects of lesion volume (*F*(1, 53) = 1.31, *p* = 0.26), scanner (*F*(3, 57) = 2.05, *p* = 0.12), age (*F*(1, 53) = 0.01, *p* = 0.94) and sex (*F*(1, 53) = 0.51, *p* = 0.48) were all nonsignificant.

**Table 2.** Mean normalized perfusion (and standard error) in left hemisphere perilesional rings and their right hemisphere homologues for the treatment and no-treatment groups at the two time points

Group	Time	Left hemisphere			Right hemisphere		
		0–6 mm	6–12 mm	12–18 mm	0–6 mm	6–12 mm	12–18 mm
Treatment (*n* = 45)	Baseline (pre)	0.69 (0.03)	0.82 (0.03)	0.86 (0.03)	1.01 (0.03)	0.94 (0.02)	0.95 (0.02)
	3-months (post)	0.71 (0.02)	0.85 (0.02)	0.90 (0.02)	1.02 (0.02)	0.96 (0.02)	0.96 (0.02)
	*change*	0.02^ns^	0.03^ns^	0.04^ns^	0.01^ns^	0.02^ns^	0.01^ns^

No-treatment (*n* = 16)	Baseline	0.68 (0.03)	0.82 (0.03)	0.86 (0.03)	0.96 (0.04)	0.93 (0.03)	0.94 (0.02)
	3-months	0.70 (0.04)	0.83 (0.03)	0.86 (0.02)	1.00 (0.05)	0.96 (0.03)	0.95 (0.02)
	*change*	0.02^ns^	0.01^ns^	0.00^ns^	0.04^ns^	0.03^ns^	0.01^ns^

*Note*. Perfusion change scores (3-months – baseline) were all non-significant. ^ns^ = non-significant.

While the overall pattern was therefore consistent with the results of experiment 1, there were no significant main effects or interactions with Group (treatment vs. control): Group (*F*(1, 53) = 0.26, *p* = 0.61), Group × Time (*F*(1, 59) = 0.06, *p* = 0.81), Group × Hemisphere (*F*(1, 59) = 0.02, *p* = 0.90), Group × Hemisphere × Time (*F*(1, 531) = 2.7, *p* = 0.10), Group × Ring (*F*(2, 531) = 1.26, *p* = 0.28), Group × Time × Ring (*F*(2, 531) = 0.24, *p* = 0.78), Group × Hemisphere × Ring (*F*(2, 531) = 0.97, *p* = 0.38), and Group × Hemisphere × Time × Ring (*F*(2, 531) = 0.14, *p* = 0.87). Also not significant were interactions of Time × Ring (*F*(2, 531) = 0.22, *p* = 0.80) and Hemisphere × Time × Ring (*F*(2, 531) = 0.14, *p* = 0.87). In addition, follow-up contrasts to examine changes in perfusion over time revealed no significant effect of Time in any perilesional ring in either hemisphere, for both the treatment and the control group (all *q*s > 0.96).

Despite the finding of no evidence of a change in perilesional perfusion over time in either the treatment or the control group, the treatment group did show a significant post-treatment gain in language performance: post- minus pre- difference score against 0: *M* = 0.28, *SD* = 0.17; *t*_0_(44) = 11.1, *p* < 0.0001; Hedge’s *g*_*av*_ effect size: 0.95, a large effect (Lakens, 2013). There was no change in performance for the control group (*M* = 0.02, *SD* = 0.06; *t*_0_(15) = 1.03, *p* = 0.32; Hedge’s *g*_*av*_ effect size: 0.07; only a small, nonsignificant effect). However, there was no relationship between their treatment gain (post-treatment – pre-treatment difference scores) and their pre-treatment perfusion, in the left hemisphere 0–6 mm perilesional region (*B* = 0.01, *t*(37) = 0.08, *q* = 0.95) or the 6–12 mm (*B* = 0.12, *t*(37) = 0.8, *q* = 0.93) or 12–18 mm rings (*B* = 0.11, *t*(37) = 0.75, *q* = 0.93), adjusted for (square root transformed) lesion volume, scanner, age, and sex. The same analyses in the right hemisphere were not significant in the 0–6 mm homologue ring (*B* = −0.03, *t*(37) = 0.24, *q* = 0.95), the 6–12 mm ring (*B* = −0.04, *t*(37) = 0.21, *q* = 0.95), and the 12–18 mm ring (*B* = 0.01, *t*(37) = 0.06, *q* = 0.95). Likewise, pre-treatment perfusion did not predict post-treatment test scores in any perilesional ring in the LH or in their RH homologues (all *q*s > 0.92).

To examine the possibility that different treatment protocols may have been more or less effective, we repeated the analyses examining perfusion changes over time, but including participant Testing site and its interactions with Group, Ring, Hemisphere, and Time. For these analyses we included age, sex, and (square root) lesion volume as predictor variables, but excluded scanner, given its collinearity with testing site. With respect to the random effects, including Testing site as a random effect did not improve model fit, so only Participant, Hemisphere, and Time were included as random effects, as above. The results revealed no significant effects or interactions with testing Site and Time (main effect of site: *p* = 0.52; Site × Time interaction: *p* = 0.80; Group × Site × Time: *p* = 0.67; Hemisphere × Site × Time: *p* = 0.59; Group × Hemisphere × Site × Time: *p* = 0.29; Ring × Site × Time: *p* = 0.54; Group × Ring × Site × Time: *p* = 0.12; Hemisphere × Ring × Site × Time: *p* = 0.99; Group × Hemisphere × Ring × Site × Time: *p* = 0.95). Follow-up contrasts to examine changes in perfusion over time revealed no significant effect of Time in any perilesional ring in either hemisphere, for both the treatment and the control group, at any of the three sites (all *q*s > 0.97).

### Discussion

In experiment 2 we looked at whether perfusion in perilesional regions changed in response to behavioral interventions targeting each person’s diagnosed language impairment (agrammatism, anomia, dysgraphia). Finally, we also examined whether pre-treatment perfusion levels in these regions might predict either the response to treatment or post-treatment language performance.

**Figure 3.**
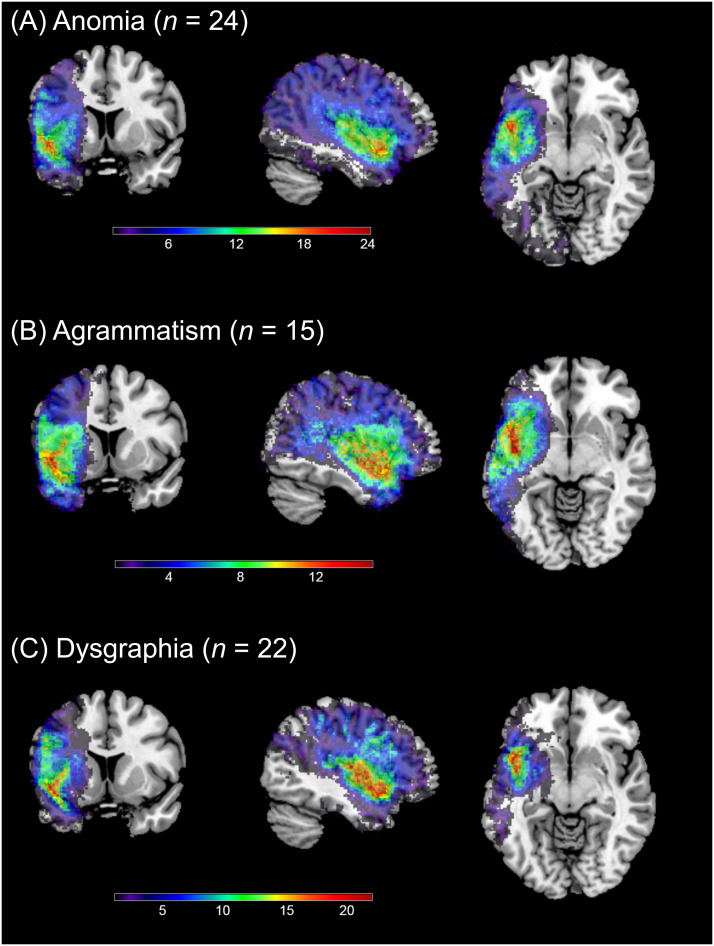
Coronal (left), sagittal (mid), and axial (right) images showing lesion overlap for 61 participants in experiment 2, separately for each site. The sagittal view shows the left hemisphere viewed from the midline.

We did not observe any change in resting state perfusion in response to our behavioral treatment interventions, despite large improvements in language performance on our treatment measures. These results are consistent with the one prior study that examined perfusion changes in perilesional rings (Boukrina et al., 2019). These null findings did not reflect the fact that we combined data across three testing sites, where participants were given different treatment protocols and may have had different lesion profiles. First, the lesion locations do not appear to be markedly different across sites (Figure 3). Moreover, in our analyses we found no interactions between perfusion changes over time with site, and no significant changes over time for any of our ROIs at any site. Thus it seems that the overall null finding reflects consistent results across sites.

Our finding that perfusion did not change over time is unlikely to reflect between-participant variability due to scanner, lesion volume, or perfusion modifiers, as we followed the same testing and data processing and analysis procedures as in experiment 1 (see above). These findings are also unlikely to reflect uncontrolled variability across sessions. Participants were scanned at the same time of day across sessions, and were asked to maintain their normal caffeine intake for each session. While we took no special measures to ensure that participants were positioned identically in the scanner for each session, the pads we added for their comfort and the tight fit of the head coils allowed for very little variation on this score. To further attempt to quantify the variation across sessions, we computed the within-subject coefficient of variation (wsCV) over the 16 participants in the control group. Results indicate coefficients of variation from 9.7% to 15.9% across our perilesional rings (Table 3). The coefficient of variation was highest for the right hemisphere 0–6 mm ring (15.9%). Coefficients of variation less than 20% are considered “good” for measures of treatment effects (Quan & Shih, 1996). Thus between-session variability seemed to be low and within reasonable bounds for us to have been able to detect a treatment effect on perfusion.

**Table 3.** Within-subject coefficient of variation (wsCV) for perilesional perfusion values

Perilesional ring	Left hemisphere	Right hemisphere
0–6 mm	11.8%	15.9%
6–12 mm	10.9%	11.4%
12–18 mm	10.3%	9.7%

These estimates of wsCV also provide an estimate of test–retest reliability for our measure. This is important to establish, because paradigms with good reliability are better suited to detect systematic changes over time (e.g., in response to treatment), whereas paradigms with poor reliability may be unsuited to detect such systematic changes. Previous findings with healthy and cognitively impaired adults indicate high test–retest reliability of pCASL sequences at intervals as long as 4 weeks, using intra-class correlation coefficients (Almeida et al., 2018; Kilroy et al., 2014). However, to our knowledge no prior study has examined perfusion reliability in any population at an interval as long as 3 months. While our estimates of wsCV indicated good reliability, they might nevertheless be improved. One possibility to improve reliability might be to use multiple post-labeling delay times and 3D imaging sequences (Kilroy et al., 2014), though we leave this investigation for future work.

We also found that baseline levels of resting state perfusion in our perilesional rings did not predict responsiveness to treatment, nor did baseline resting state perfusion in these rings predict post-treatment test scores. These results are consistent with one prior study that also found no relation between baseline perfusion in perilesional rings and treatment response (Fridriksson et al., 2012), but are at odds with another that reports a negative effect between treatment response and perfusion in RH regions homologous to LH perilesional rings (Boukrina et al., 2019). We saw no relation between RH perfusion and treatment, despite our finding of abnormally increased perfusion in our RH rings.

One possibility for these findings is that our treatments did not lead to a change in vascular physiology that affects resting state perfusion in the perilesional rings that we examined. This seems clear from the distinct lack of a perfusion change over time. Nevertheless, our treatments were effective at improving language. It may be that perilesional tissue was re-engaged, but only during active language processing. If so, it may be that responsiveness to our language treatments in these perilesional rings would be better predicted by measures of CBF that capture local vascular dilations that occur during a task, such as the time-to-peak of the hemodynamic response function (Bonakdarpour et al., 2007, 2015; Thompson et al., 2010). Such measures might reflect improved neural activity or metabolism (Ogoh et al., 2014). A second possibility is that our participants had already undergone some degree of functional reorganization prior to our treatment interventions. If so, regions other than the perilesional tissue may have been compensating for the impaired functions. Our treatments could therefore have improved the efficiency of such compensation by these other regions, potentially leading to changes in resting state perfusion outside of the perilesional regions we examined here. These issues, and the discrepancy between our findings and Boukrina et al.’s (2019) prior findings in the right hemisphere, require further research.

## CONCLUSIONS

In sum, we report four findings related to cerebral perfusion in chronic aphasia subsequent to stroke. First, we find a gradient pattern of perilesional hypoperfusion, with decreasing perfusion with increasing nearness to the lesion. Second, we find an island of relatively increased perfusion in the right (contralesional) hemisphere, that echoes the lesion and, to a lesser degree, the most proximal perilesional tissue. The behavioral treatments that our participants were given did effectively improve their language performance on the treatment materials. However, our behavioral treatment interventions did not entail any concomitant changes in perfusion in our perilesional rings or their RH homologue regions. Finally, abnormal baseline perfusion levels in these perilesional rings or their RH homologues did not predict either language improvement or post-treatment language performance.

## ACKNOWLEDGMENTS

This work was supported by the NIH-NIDCD, Clinical Research Center Grant, P50DC012283 (PI: Cynthia K. Thompson), and was completed while the first author (Matthew Walenski) was at Northwestern University. The authors wish to thank Xue Wang, Elena Barbieri, Sladjana Lukic, and Brianne Dougherty for assistance with data collection and analysis, and to thank Neda Mohammadi for assistance with literature review.

## FUNDING INFORMATION

Cynthia K. Thompson, National Institute on Deafness and Other Communication Disorders (https://dx.doi.org/10.13039/100000055), Award ID: P50DC012283.

## AUTHOR CONTRIBUTIONS

**Matthew Walenski**: Conceptualization; Formal analysis; Visualization; Writing – original draft; Writing – review and editing. **Yufen Chen**: Data curation; Methodology; Visualization; Writing – original draft; Writing – review and editing. **Kaitlyn A. Litcofsky**: Data curation. **David Caplan**: Conceptualization; Funding acquisition; Writing – review and editing. **Swathi Kiran**: Conceptualization; Funding acquisition; Project administration; Supervision. **Brenda Rapp**: Conceptualization; Funding acquisition; Project administration; Supervision. **Todd B. Parrish**: Conceptualization; Funding acquisition; Project administration; Supervision; Writing – review and editing. **Cynthia K. Thompson**: Conceptualization; Funding acquisition; Project administration; Supervision; Writing – review and editing.

## TECHNICAL TERMS

Anomia: The impaired ability to produce or recognize the spoken or written names of an object.
Agrammatism: Speech production that is characterized by short, syntactically impoverished utterances with frequent pauses and the omission of function words, along with an impaired ability to produce and comprehend syntactically complex sentences.
Dysgraphia: The impaired ability to produce written language.
